# Thyroid papillary carcinoma arising in ectopic thyroid tissue within a neck branchial cyst

**DOI:** 10.1186/1477-7819-4-24

**Published:** 2006-05-03

**Authors:** Angela Fumarola, Pierpaolo Trimboli, Rossana Cavaliere, Iolanda Coletta, Alessandra Veltri, Agnese Di Fiore, Antonio Ciardi, Francesca Piccirilli

**Affiliations:** 1 Department of Experimental Medicine and Pathology, Chair of Endocrinology, University of Rome "La Sapienza", Rome, Italy; 2 Department of Surgery "P. Valdoni", University of Rome "La Sapienza", Rome, Italy

## Abstract

**Background:**

Thyroid gland derives from one median anlage at the base of the tongue, and from the two fourth branchial pouches. A number of anomalies may occur during their migration. These can be in form of ectopic tissues, which are frequently found along the course of thyroglossal duct and rarely in other sites, many of these may develop same diseases as the thyroid gland.

**Case presentation:**

A 36-years-old female presented with a 3 month history of left side neck mass. The mass disappeared following aspiration of brown colored fluid, which on cytological examination showed cells with nuclear irregularities that warranted the resection of the lesion. The histology demonstrated a thyroid papillary carcinoma arising within the branchial cyst. Thereafter, the patient underwent a total thyroidectomy with central lymph nodes dissection. Histology showed a multifocal papillary carcinoma with central lymph nodes metastases. Only four cases of primary thyroid carcinomas in neck branchial cyst have been described so far.

**Conclusion:**

In a lateral cystic neck mass, although rare, occurrence of ectopic thyroid tissue and presence of a papillary thyroid carcinoma should be kept in mind.

## Background

Human thyroid gland derives mainly from one median anlage, which develops from invagination in the floor of the primitive pharynx, at the base of the tongue. This anlage, during its maturation, migrates downward, along the transient thyroglossal duct, which undergoes atrophy prior the definitive thyroid formation. At the same time, lateral anlages of two fourth branchial pouches share the development the gland. From the last, two superior parathyroid glands and the lateral thyroid are derived. The ultimobranchial bodies originate from the fifth branchial pouches and migrate downward on each side of the neck. From these develop the parafollicular C-cells, which make calcitonin. A number of anomalies may develop either from the gland or from parts of it during this process. These ectopic tissues may develop the same diseases as the thyroid gland [1].

## Case presentation

**Figure 1:**
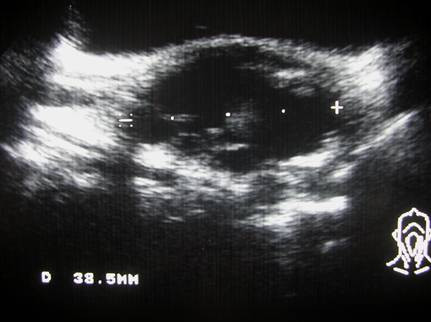
Neck ultrasound evaluation showed a mixed solid and cystic lesion with septations, and irregular and thick margins in lateral region.

**Figure 2:**
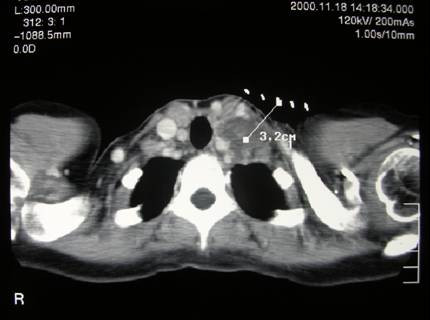
Computed tomography showing a low density lesion with irregular and thick margins in lateral neck region.

**Figure 3:**
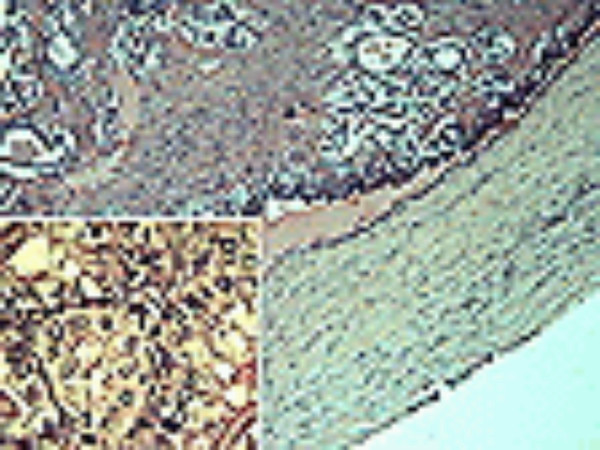
Photomicrograph show the thick wall of the cyst with adiacent area of papillary carcinoma (follicular variant) (HE 50×), with the nuclear features of the lesion (inset, HE, 400×).

**Figure 4:**
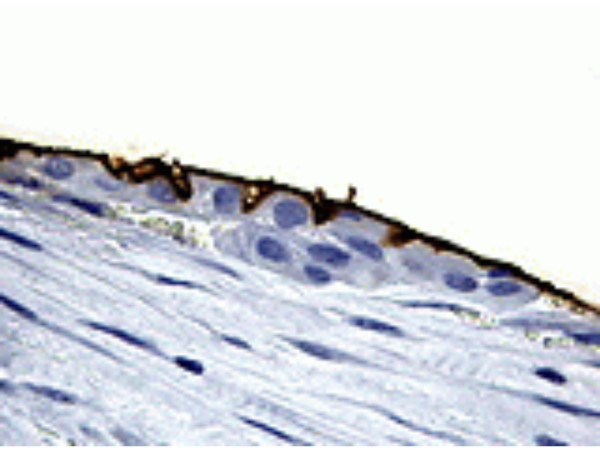
Section of the cyst wall showing the lining composed of cuboidal cells with positive staining for thyroglobulin at the apical portion of cytoplasm. (DAB, 250×).

A 36-years-old female presented with a 3-month history of a hard, mobile and painless left side neck mass. No other masses were palpable. The ultrasound (US) evaluation showed a 38.5 mm solitary cystic lesion with irregular margins in the left lateral cervical region that lacked a vascular signal (Figure 1). Thyroid US showed a gland with an inhomogeneous echostructure but devoid of any focal lesion. A computed tomography scan (CT) the neck lesion showed a low density, irregular-circumscribed mass measuring 35 mm (Figure 2). The mass disappeared following a CT-guided aspiration of 15 ml of chocolate brown colored fluid. This fluid, on fine needle aspiration cytology (FNAC) examination showed an amorphous and hematic material and cells with nuclear irregularities that warranted the resection of the lesion. The histology of the resected lesion demonstrated a thyroid papillary carcinoma arising within the multiloculated neck branchial cyst (Figure 3). The non neoplastic cystic spaces were lined by a monolayer of cuboical cells with granular eosinophilic cytoplasm and central enlarged nuclei. At immunohistochemistry for thyroglobulin, these cells showed positive staining of the apical portion of the cytoplasm (Figure 4). After one month, the patient underwent a total thyroidectomy with central and lateral compartment lymph nodes dissection. Histology showed a multifocal papillary carcinoma. The dominant nodule, with extra-capsular invasion, had a size 4 mm. Furthermore, 20 out of 25 neck lymph nodes removed (pericarotid, perireccurrent, jugular, central compartment) showed metastases. Diagnostic whole body scan (with 5 mCi of ^131^I) showed hyper fixation (star-like image) in central region of neck. A dose of 100 mCi of ^131^I was administrated in hypothyroidism (TSH 80 mU/ml, thyroglobulin 32 ng/ml, anti-thyroglobulin antibody negative) and whole body scan showed hyper fixation in mediastinal, median cervical region and diffuse fixation in lungs (pT3 N1b M1).

## Discussion

Ectopic thyroid tissue is reported in 7% of adults [2] and is frequently found along the course of thyroglossal duct or around the two lobes of the gland. Other possible sides of ectopic localization are anterior tongue, larynx, trachea, esophagus, mediastinum, pericardium, diaphragm and, rarely, neck branchial cyst. Previous reports showed that ectopic thyroid tissue may present metastasis from thyroid carcinoma, and very rarely it may harbour a primary thyroid carcinoma. Of the latter, about one hundred cases have been so far described in literature [3]. Most of them have been shown to occur in the thyroglossal duct, 1% out of all thyroglossal cysts carcinomas are papillary carcinomas [3,4]. Only four cases of primary thyroid carcinomas arising in neck branchial cyst have been described by Balasubramaniam *et al*., in 1992 [5], Jadusingh *et al*., in 1996 [6], Matsumoto *et al*., 1999 [7], and Cappellani *et al*., 2004 [8]. The present case is the fifth documented case.

In appearance of a lateral neck cyst, the possibility of malignancy of the lesion, the occurrence of a branchial cyst including thyroid ectopic tissue, and the presence of a primary thyroid carcinoma arising in this lesion, have to be taken into account [9,10]. Since US, CT and FNAC are not able to give diagnostic information in the present case, this suggests that neck branchial cyst evaluation should be included in the analysis of all risk-factors and clinical evaluation is to be applied in the management of thyroid nodules (i.e. external neck irradiation, age under 20 or over 70, male sex). Because of the cystic structure of the lesion, FNAC may not be diagnostic. Measurement of thyroglobulin protein and mRNA in needle wash out, although are able to recognize the presence of ectopic thyroid tissue, is not able to discriminate between benign and malignant lesion. In such situation, only the histological evaluation following surgery may provide a definitive diagnosis of the lesion.

## Conclusion

In evaluation of a lateral neck cyst, the occurrence of a branchial cyst including thyroid ectopic tissue and the presence of a thyroid carcinoma arising in this lesion should be taken into account. There are no clinical, biochemical, or imaging parameters exist that may assist in determining the nature of the lesion, and histological exam always is required for definitive diagnosis.

## Competing interests

The author(s) declare that they have no competing interests.

## Authors' contributions

**IC**, **ADF**, **AV**,: Acquisition of data, drafting of manuscript, **PT**, **RC**,: Study conception and design, **AF**: Analysis and interpretation of data, **FP&AC**,: critical revision and supervision. All authors read and approved the final manuscript.
